# Standardized Usage of Electronic Patient-Reported Outcome Measurements is Time-Efficient and Feasible

**DOI:** 10.3390/jpm14090986

**Published:** 2024-09-17

**Authors:** Thilo Khakzad, Michael Putzier, Alexander Bartschke, Rasim Atakan Poyraz, Nima Taheri

**Affiliations:** 1 Center for Musculoskeletal Surgery, Charité—Universitätsmedizin Berlin, Corporate Member of Freie Universität Berlin, Humboldt-Universität zu Berlin, 10117 Berlin, Germany; thilo.khakzad@charite.de (T.K.); michael.putzier@charite.de (M.P.); 2 Core Facility Digital Medicine and Interoperability, Berlin Institute of Health at Charité—Universitätsmedizin Berlin, 10117 Berlin, Germany; alexander.bartschke@bih-charite.de (A.B.); rasim-atakan.poyraz@charite.de (R.A.P.)

**Keywords:** digitization, patient-reported outcome measurements, PROMs, ePROMs

## Abstract

(1) Background: Digitization is of the utmost importance in improving the transfer of medical data. In order to emphasize the need for the greater implementation of digital solutions, we compared analog PROMs (aPROMs) to electronic PROMs (ePROMs) to emphasize the time benefits for clinical everyday life. (2) Methods: This prospective, observational study compared the evaluation of SF-36 in patients between 18 and 80 years old with musculoskeletal pathologies. We performed an age-independent and age-dependent analysis. (3) Results: After the import of aPROMs data, ePROMs took significantly less time (11.97 ± 3.00 min vs. 9.41 ± 3.12 min, *p* = 0.002, d = 0.797). There were no significant differences associated with age for aPROMs (7.23 ± 2.57 min vs. 8.38 ± 2.71 min, *p* = 0.061, d = −0.607) or ePROMs (8.72 ± 2.19 min vs. 10.09 ± 3.80 min, *p* = 0.130, d = −0.436), respectively. (4) Conclusions: This study indicates that ePROMs are a time-feasible method for collecting data to guide patient-personalized treatment approaches.

## 1. Introduction

The need for advances regarding digitization in medicine is omnipresent. The goal is to improve the local, regional, national and international transfer of medical data in order to supply every patient with the best possible treatment. The necessity of digitization stems from the key components: rapid technological development in data processing, an everimproving understanding of human biology and growing patient sovereignty with the growing desire for transparent healthcare [1].

With the rise of technology, the need for evidence-based evaluations is increasing by the minute. Therefore, usage of patient-reported outcome measurements (PROMs) has been increasing in studies, particularly in those with high-evidence recommendations [2]. So-called electronic PROMs (ePROMs) are routinely applied in different sections of medicine, e.g., in oncology, rheumatology, radiation therapy, internal medicine and surgery [2,3,4,5]. The goal of the implementation of PROMs within clinical settings is to compare health services, identify the strengths and weaknesses of healthcare delivery, improve clinical decisions and empower patients’ choices [6].

The utilization of PROMs is not a novelty in medical science. Emerging evidence suggests that ePROMs are generally preferred and more accepted by patients than aPROMs [7]. This is accompanied by reduced costs, faster completion times and improved data quality and response rates [7,8].

The analog data collection and management of paper-based PROMs can be time- and resource-consuming. Digital platforms could streamline data collection, automate scoring, save time and costs and improve efficiency. Participants see ePROMs as less of a barrier and their time for completion is more accepted [8]. Studies by Bliven et al. and Ali et al. have confirmed significantly reduced completion times for ePROMs [9,10]. Touvier et al., in a study involving 500,000 subjects, demonstrated that financial costs could be reduced from EUR 4,965,833 (EUR 9.94/subject) to EUR 150,000 (EUR 0.3/subject) [8]. Moreover, the average human resource time required is reduced from 25 min for aPROMs to 9.5 min with ePROMs [11].

Patient engagement and compliance are critical considerations. In some cases, single PROMs can take up to 30 min. Evidence indicates a clear patient preference for ePROMs over aPROMs [12,13]. This is especially important when assessing complex pathologies—such as psychosomatic ones—where multiple PROMs are evaluated. Interactive and user-friendly interfaces improve both the short- and long-term compliance of the patient [12].

The data’s accuracy and reliability may be more susceptible to errors during data entry, transcription and scoring in aPROMs. Multiple studies have indicated less missing or incomplete data with ePROMs [8,11,13,14]. Also, participants tend to give more detailed information on open-ended questions when using ePROMs [15]. The digitization of PROMs not only streamlines data collection but also enhances the accuracy and reliability of the information gathered, thereby directly supporting personalized treatment approaches [16]. Furthermore, the digitization of PROMs facilitates the integration of data into broader healthcare. This enables more comprehensive analyses and better-informed clinical decisions [6]. 

In order to put emphasis on the need for ePROMs, we conducted a prospective study in which we evaluated the time benefit that emerges when using ePROMs in comparison to paper aPROMs. We hypothesize that ePROMs are significantly faster to complete, regardless of age group. This study aims to demonstrate that ePROMs are not only more efficient but also integral to advancing personalized, evidence-based care.

## 2. Materials and Methods

The study was approved by a local ethics committee (EA1/189/23). Patients aged 18–80 years, presenting at our clinic for various musculoskeletal pathologies (e.g., coxarthrosis, gonarthrosis, ligamentous knee injuries, deformative degenerative spine pathologies), were invited to participate. Informed consent was obtained from all participants.

Before the start of the study, the number of patients to be included was determined. The Institute of Biometry and Clinical Epidemiology at Charité—Universitätsmedizin Berlin provided consultations for this purpose. The consultation aimed to prove our primary hypothesis. We aimed to demonstrate that the electronic collection of the SF-36 is better than analog collection followed by electronic data entry by staff. 

The SF-36, a widely used self-reported questionnaire, was employed to measure health-related quality of life (QoL). This generic instrument assesses both physical and mental health across eight domains: physical functioning, role limitations due to physical problems, bodily pain, general health, vitality, social functioning, role limitations due to emotional problems, and mental health.

Semantic interoperability is essential for ensuring that PROMs data can be consistently understood and interpreted across different healthcare systems, applications and platforms. This requires the use of standardized terminologies, coding systems and ontologies to define and represent PROMs concepts in a universally comprehensible manner.

The adoption of common data elements (CDEs) for PROMs is a key aspect of achieving semantic interoperability. CDEs define standardized data elements, such as PROMs questions and response options, ensuring uniformity and compatibility across different studies and healthcare settings. This standardization facilitates data integration and comparative analyses, enhancing the reliability and validity of PROMs data [17]. Furthermore, semantic interoperability also supports data aggregation and the secondary use of PROMs data. Researchers and healthcare stakeholders can combine PROMs data from various sources to conduct large-scale analyses, identify trends and generate evidence to inform clinical decision-making and healthcare policy [18]. Standardized PROMs terminologies, such as the Systematized Nomenclature of Medicine—Clinical Terms (SNOMED CT) and Logical Observation Identifiers Names and Codes (LOINC), are essential for achieving semantic interoperability [19]. These terminologies provide a shared vocabulary for describing PROMs concepts, ensuring consistency in data representation and facilitating data exchange between different healthcare information systems. This study was conducted by using the Unity Platform of Raylytic Firm (Leipzig, Saxony, Germany) to enable us to capture data simultaneously from interoperable SF-36 PROMs for further analysis.

To address the potential bias related to patient familiarity with digital devices, participants were asked about their proficiency with digital technology before being included in the study.

During the analog measurement, a study supervisor accompanied each patient. The supervisor started the timer at the beginning of the questionnaire and recorded the time until it was completed. 

Afterwards, the data were manually evaluated. The time required for evaluation was also recorded. The collected data were imported into the electronic patient record in the clinical interface. 

For the digital measurement, the time required from logging in to the completion of the questionnaire was similarly recorded. 

In both cases, the study supervisor did not stop the time in cases of questions regarding the PROMs by the patient. Therefore, the time measured would represent the actual time needed to fully understand and evaluate the PROMs.

Data analysis was performed using SPSS Version 27 (IBM Corp. Released 2020. IBM SPSS Statistics for Windows, Version 27.0. Armonk, NY, USA: IBM Corp). The Kolmogorov–Smirnov test was performed in order to test for the normal distribution of the values. We compared the time for completion of aPROMs and ePROMs measurements using an unpaired t-test. Furthermore, we added the time for completion of the data, as well as its import into the clinical interface, for the analog measurement and compared it again to the time needed for ePROMs using an unpaired t-test. In order to assess the influence of age on the time needed, we performed an unpaired t-test. Effect sizes were calculated as Cohen’s d. A Cohen’s d of 0.2 was considered a small effect size, 0.5 as moderate and 0.8 as a large effect size. 

## 3. Results

### 3.1. Comparison of aPROMs vs. ePROMs

**Figure 1:**
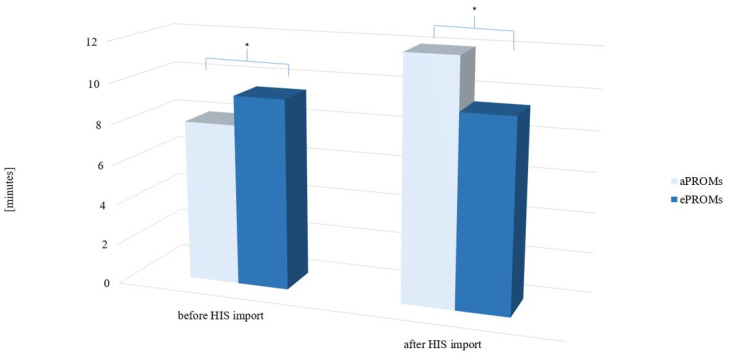
Comparison of time needed for completion of aPROMs and ePROMs before and after hospital information system (HIS) import; aPROMs = analog PROMs, ePROMs = digital PROMs; * = *p* < 0.05.

Participants in the aPROMs group (n = 28) completed the evaluation significantly faster than those in the ePROMs group (n = 28; 7.97 ± 2.71 min vs. 9.41 ± 3.12 min, *p* = 0.035, d = −0.492; Figure 1).

After accounting for the time required to import the data into the hospital information system (HIS), participants completing aPROMs performed significantly worse than those using ePROMs (11.97 ± 3.00 min vs. 9.41 ± 3.12 min, *p* = 0.002, d = 0.797).

Participants were further categorized into two age groups: the younger age group (18–49 years), with 14 participants for each modality, and the older age group (50–80 years), also with 14 participants for each modality.

### 3.2. Influence of Age on Evaluation of aPROMs and ePROMs

There were no significant differences in the time needed for the evaluation of aPROMs before the HIS import that were associated with age (7.23 ± 2.57 min vs. 8.38 ± 2.71 min, *p* = 0.061, d = −0.607). Furthermore, there were no significant differences associated with age considering the evaluation of ePROMs (8.72 ± 2.19 min vs. 10.09 ± 3.80 min, *p* = 0.130, d = −0.436).

### 3.3. Data Accuracy

In the aPROMs group, 4 out of 28 participants (14.29%) made errors in their evaluation of the SF-36, compared to none in the ePROMs group (0.00%). This difference is statistically significant (*p* = 0.019; d = 0.567).

## 4. Discussion

We were able to confirm our primary hypothesis and demonstrate that the evaluation of ePROMs is significantly superior to the collection of aPROMs in both the time the evaluation takes and the accuracy of the data. In contrast to other study groups, we were not able to show a significant influence of age. Therefore, ePROMs can significantly reduce the time needed for the evaluation of PROMs. Furthermore, they improve data accuracy. These benefits are independent of age and further emphasize the need for the digitization of PROMs to streamline personalized medicine.

The evidence regarding the time efficiency of ePROMs is controversial. Walther et al. could not show any significant differences between these modalities [20]. Schmidt M et al. reports time reductions of 59–69% by the digital entry of data [21]. Further, Shah KB and Greenwood likewise showed no significant differences in the time saved [22,23], whereas Salaffi et al. and Hollen et al. reported significant time advantages for ePROMs [24,25]. Similar to the research group around Fleischmann et al., we were able to show a reduction of circa 22% after the complete import of analog data into the clinical interface [26]. The time saved by using ePROMs can positively impact clinical workflows [24,25,26,27]. This might include reducing the burden on healthcare providers, allowing more time for interactions and enabling quicker data processing and decision-making [24,25,26,27,28,29]. Faster completion times could lead to higher patient satisfaction, which is important for compliance and overall treatment outcomes [7,29]. This is also of interest in. Previous study groups showed a significant cost reduction and reduction in the human resources needed when using ePROMs for large-scale studies [8,11]. 

Further, we were able to refute the widely held belief that aPROMs are much more prone to data errors, significantly degrading the quality of their data [14]. Of the participants in the analog arm, 29% filled in the questionnaire incorrectly, mainly by giving duplicate answers to express subjective indecision. In the context of ePROMs, this behavior is prevented by built-in control algorithms and thus—in theory—reduced to zero. This result is consistent with work by Engan et al., Rada et al., Touvier et al., Smith Mj et al. and Kongsverd et al., all of which demonstrate significant increases in data accuracy when ePROMs are collected [7,10,12,13,14]. Thus, our error rate was also much higher than the ratios described in the research, with 5% errors for aPROMs vs. 1% for ePROMs [17,26]. Data accuracy is an essential part of evidence-based medicine. A reduction in data errors leads to better clinical decision-making and, in the future, could potentially streamline personalized treatment plans.

In terms of age, we did not find significant differences in the duration required to complete the PROMs. This finding is significant because it demonstrates the universal application of digitized healthcare applications. Robotham et al. questioned the digital skills of the older generation, for understandable reasons. The evidence on this is thin [27]. The research group led by Fleischmann et al. also showed that there was no effect of age on the time required to complete analog and digital questionnaires in a regression analysis [28]. This further solidifies the need for ePROMs, as a time-efficient tool, to be implemented into daily clinical routine. Their widespread adoption can lead to more informed decision-making in various patient groups. Recent studies have shown their impact in gathering feedback regarding the side effects of radiation therapy, with high rates of patient satisfaction [29]. Furthermore, this can improve the relationship between patients and their clinical team in various fields of medicine, with the most significant data coming from oncology and nephrology patients [30].

The lack of digitalization poses a significant barrier that must be overcome to fully harness the advantages of PROMs data, ensuring their seamless integration into clinical processes to provide healthcare providers and physicians with real-time, relevant information and making PROMs data usable for AI applications. For example, ePROMs can be used to gather automated evidence of the development of complications after therapeutic interventions [31,32]. Thereby, the patient’s sovereignty is displayed by a graphical representation of his therapy progress and his compliance is increased [33,34]. ePROMs can also be integrated with other digital health tools like electronic health records or telemedicine platforms. This further generates seamless workflows for both patients and healthcare providers [35,36]. Furthermore, ePROMs could be a valuable source of data for AI applications, enabling predictive analytics and treatment recommendations [37].

Future studies should focus on including patients from various medical fields. By analyzing different medical areas, the impact of ePROMs could potentially be of even more significance. Furthermore, evaluating the financial impact of the transition from aPROMs to ePROMs should be a subject of interest as it translates to the feasibility of the transition [38,39]. Pronk et al. were able to show that the manual collection of PROMs would cost around EUR 5.55–5.98 per surgical procedure [39]. 

The study is limited by the potential selection bias introduced by including only patients capable of handling electronic devices. This criterion likely excluded individuals with cognitive impairments, physical disabilities, or lower technological literacy, thereby skewing the sample towards a healthier population. Consequently, the generalizability of our findings may be limited, as the results may not accurately represent the broader patient population. Lastly, the generalizability of our findings is compromised by the small number of participants. Future studies should include more patients from various medical fields.

In summary, we were able to show a significant reduction in the time required to complete PROMs in a digital format. We expect that the generalized use of ePROMs to improve patient outcomes will be implemented in the coming years with close adherence to local privacy regulations.

## 5. Conclusions

Within our study, we were able to demonstrate that the evaluation of ePROMs is significantly faster than that of aPROMs after their import into the HIS. Additionally, we showed that ePROMs are a feasible method for the evaluation of subjective health parameters across all age groups. 

As modern healthcare increasingly prioritizes efficiency and individualized patient care, the systematic evaluation of PROMs data becomes essential. These data enable evidence-based medicine to better characterize individual patients, leading to more personalized therapeutic approaches. The integration of ePROMs into HIS not only streamlines clinical workflows but also enhances the precision of patient care by facilitating the digitization and personalization of health data. Looking ahead, the broader adoption of ePROMs has the potential to further advance personalized medicine, improving patient outcomes by providing timely and accurate insights into patient health, thereby supporting more informed clinical decisions.

## Data Availability

The datasets generated and analyzed during the current study are available from the corresponding author on reasonable request.
